# Supplementary material for: Evaluation of serum uric acid and liver function tests among pregnant women with and without preeclampsia at the University of Gondar Comprehensive Specialized Hospital, Northwest Ethiopia
Source: PLoS One. 2022 Aug 4;17(8):e0272165. doi: 10.1371/journal.pone.0272165 (PMC9352010; doi:10.1371/journal.pone.0272165)
Supplement: Attachment — Submitted filename: PLOS ONE_Review.docx [file pone.0272165.s001.docx]

Review:

In this cross-sectional study, Dr. Hassen et al aimed to evaluate the utility of levels of serum uric acid and liver function tests/transaminases as early biomarkers of pre-eclampsia-related end organ damage. Both elevated serum uric acid and liver function tests/transaminases are potential laboratory findings in pre-eclampsia. Although the findings are not necessarily novel, the text is clear and easy to read, but I do have some suggestions as outlined below. There are also inconsistencies in some of the authors’ statements, which need to be corrected/modified.

Abstract:

Line 11: Pre-eclampsia can be described as new-onset hypertension…

Line 12: …or ANY indication…

Line 13: Liver and kidney dysfunction…

Line 14: …common MANIFESTATIONS of end-organ damage…

Line 15: …most common cause of LIVER and KIDNEY dysfunction…(Please change all “renal” terms to “kidney” instead.)

Line 15: Pre-eclampsia is the most common cause of liver and kidney dysfunction – *in what patient population?* – due to hypoxia and endothelial dysfunction.

Line 17: Hyperuricemia indicates kidney dysfunction…

Line 18: …severity of pre-eclampsia; therefore, the objective of this study is to evaluate the utility of the levels of serum uric acid and liver function tests [alanine aminotransferase (ALT) and aspartate aminotransferase (AST)] as early biomarkers…

Line 20: An institution-based comparative cross-sectional study design…

Line 21: …conducted, …

Line 22: The parameters measured were levels of serum uric acid and liver function tests.

Line 24: There were statistically significant differences in the mean serum uric acid, ALT, and AST levels …

Line 26: There were no statistically significant differences in the mean total and direct bilirubin levels…

Line 27: There were also statistically significant differences in the mean serum uric acid, ALT, and AST levels across different gestational age categories.

Line 30: Our study revealed that serum uric acid, ALT, and AST levels were HIGHER in pre-eclamptic pregnant women compared to THOSE of healthy pregnant women, and the differences were statistically significant. As such, serum uric acid and liver function tests may be considered as early biomarkers of pre-eclampsia-related end organ damage.

Background:

Line 38: Pregnancy-induced hypertension is a major cause of maternal and fetal morbidity and mortality.

Line 40: …hypertensive disorders exist, including…

Lines 43-53: *I’m not sure if these definitions should be included as they make the background longer than it should be.*

Line 54: Pre-eclampsia is defined as new-onset hypertension…

Line 55: …or ANY sign of end-organ damage…

Line 56: *Please add* – “Proteinuria, however, is not a requirement anymore to make a diagnosis of pre-eclampsia.” (Reference: PMID: 32063058)

Line 58: Disease severity varies, and it is considered severe if it is associated with one or more of the following: …

Line 59: … at least 6 hours apart, a 24-hour protein of ≥5 g, and/or other symptoms, such as…

Line 62: *Please add* – “The main underlying disease mechanism is endothelial dysfunction affecting multiple organs, including brain, liver, kidneys, and placenta.” (Reference: PMID: 32063058)

Line 66: Its global prevalence is extremely variable…

Line 69: In Africa, it occurs in 10% of pregnancies…

Line 70: Around 4 in 100 women experience PROBLEMS with high blood pressure and kidney dysfunction…

Line 75: …maternal deaths, most of which are…

Line 76: Liver and kidney dysfunction…common manifestations of end-organ damage…

Line 78: Hyperuricemia indicates kidney dysfunction because of decreased glomerular filtration, decreased tubular secretion, and/or increased proximal tubular reabsorption. (Reference: PMID: 15660336) It is also an independent risk factor for cardiovascular diseases as it has been suggested that it alters vascular function and mediates vascular inflammation. As such, hyperuricemia can perhaps predict the severity of pre-eclampsia.

Lines 81-85: Pre-eclampsia is also the most common cause of liver dysfunction in 3% of pregnancies because of microvesicular fat deposition and reduced blood flow to the liver potentially causing ischemia and periportal hemorrhage. (References: PMID 3189435 and PMID 8607495). ALT and AST are usually normal, but when they become elevated and are accompanied by abdominal pain, it almost always suggests the severe end of the disease spectrum.

Line 86: As such, this study was undertaken to evaluate the utility of levels of serum uric acid and liver function tests as early biomarkers of pre-eclampsia-related end organ damage…

Methods and Materials:

Line 91: An institution-based comparative cross-sectional study design…

Line 92: All patient had antenatal care follow-up…

Line 94: We excluded pregnant women who had…, those with a history of medication USE (eg…), and those with substance abuse (eg smoking and alcohol consumption) as these factors may affect the outcome variables.

Line 101: …to evaluate the levels of serum uric acid and liver function tests…

Line 104: …sample size calculations, like Minitab and Epi-info, …

Line 105: As an input, G-Power requires selecting appropriate test family (t-test in this case) and statistical test within test family (linear regression in this case) and specifying alpha error probability, …

Line 108: …(80%), and effect size…

Line 112: Trained nurses…

Line 113: Four laboratory technicians were oriented about blood sample collection and storage. Questionnaires were filled by face-to-face interviews with participants. Pre-pregnancy BMIs were calculated from…

Line 118: *New sentence* – “Two consecutive blood pressure readings were also measured.”

Line 122: It was then centrifuged…

Line 124: …refrigerator UNTIL…

Line 132: …to check IF there is any data inconsistency and to avoid any problem with data entry processes…

Line 138: All data were checked for cleaning and completeness. Data processing and analysis obtained from…

Line 144: …and other assumptions WERE checked before doing any statistical analysis and WERE fulfilled.

Line 147: WITH bivariable linear regression analysis, variables with…WHILE WITH multivariable linear regression analysis, variables with…

Line 152: Ethical clearance was obtained from…

Line 159: The present study recruited 102 female study participants, 51 of whom were pre-eclamptic pregnant women while 51 of whom were healthy pregnant women with gestational age above 20 weeks.

Line 161: The mean ages of pre-eclamptic patients and healthy controls were 32.9 years (range: 21-42) and 29.5 years (range: 23-35) RESPECTIVELY.

Line 162: The mean gestational ages of pre-eclamptic patients and healthy controls were 33.6 weeks (range: 22-39) and 29.3 weeks (range: 21-38) RESPECTIVELY.

Line 164: Majority of the study participants were married. I will delete the statement - “and the rest were single in both case and control groups.”

Line 166: Among the healthy controls, 41 (80.4%) were living in urban areas.

Line 176: Based on the calculation, …

Line 178: Majority of the healthy controls (94.1%) had normal BMI while the rest (5.9%) were overweight.

Line 179: None in the control group WAS underweight. Blood pressures of the study participants were recorded…

Line 186: I think it would be better to include the reference ranges for the different laboratory parameters in Table 3.

Line 188: Majority of the pre-eclamptic patients…but had normal total and direct bilirubin levels (60.8% and 62.7% respectively).

****Under paragraphs 3.3 and 3.4, the abbreviation SUA was used instead of serum uric acid. For consistency, the authors can start using the abbreviations at the start of the manuscript and make sure to spell them out on first usage and then use the abbreviations afterwards. Aside from SUA, the rule also applies to ALT and AST.*

Lines 196-199: …statistically significant difference…

Line 200: There was, however, no significant difference in the total and direct bilirubin levels…

Line 202: The age and the gestational age of the study participants were also not significantly different…

Line 204: …compared to that of healthy controls…

Line 215: …; however, there was no significant difference in the mean SUA levels between the age groups 19-25 years and ≥35 years…

Line 219: …in mean ALT levels across different age groups…

Line 221: …; however, there was no significant difference in the mean ALT levels between the age groups…

****General comments for the results sections:*

1. *The word “level” should be inserted after all these phrases “mean SUA, ALT, and AST,” i.e. mean SUA level, mean ALT level, and mean AST level.*
2. *The preposition“of” can be deleted in all sentences containing the phrase “between the age groups,” i.e. between the age groups 19-25 years and 26-34 years. There is no need for the preposition “of” in this case.*
3. *To be grammatically correct, there should be a semicolon and a comma before and after the word “however” - …; however,…*

Discussion:

Lines 303-306: *I think that these statements need not be included in the discussion. I would consider starting the discussion by summarizing your key findings.*

Line 307: In our study, majority of the pre-eclamptic patients were found…, which is in keeping with the results of a study conducted by…showing that advanced maternal age…

Line 309: Other studies conducted in Finland and Japan also demonstrated that…

Line 312: Our study also showed that the…were found to be…

Line 313: …, and there was no statistically significant difference between the two groups. This finding is in contrast in with the findings of other studies, and this difference may be due to…

Line 317: In our study, the mean serum uric acid level was significantly higher in cases compared to controls…, which is again consistent with the findings of other studies. *I would elaborate more on these findings and explain the mechanism/s behind these findings.* In addition, the mean serum ALT and AST levels were also significantly higher…

Line 323: …inflammatory response, resulting in endothelial dysfunction causing vasoconstriction and eventual liver and kidney dysfunction…

Lines 325-331: *These statements are somewhat a repetition of the statements in lines 320-324. I would combine these 2 paragraphs to be more cohesive.*

Line 332: On the other hand, our study showed that there was no significant difference in the total and direct bilirubin levels…

Line 334: Our findings are in contrast with the findings of other studies wherein there were significant increases in…

Line 336: …due to variations in the extent of…

Line 337: In pre-eclampsia, the main cause of hyperbilirubinemia is thought to be disseminated…

Line 340: In our study, there were significant differences in the mean SUA, ALT, and AST levels across the different age groups…The mean SUA level in the age group 26-34 years was 4.43 mg/dL, which then increased to 5.66 mg/dL in the age group >35 years. *Please modify the sentences after this one to the same format.*

Line 346: These findings were consistent with those of other studies, which showed that…

Line 348: Similarly, there were also significant differences in the mean SUA, ALT, and AST levels across the different gestational age groups…*Please also modify the sentences after this one to the same format as mentioned above.*

Line 357: Lastly, we performed a multivariable linear regression analysis and found a statistically significant positive association between…

Lines 362-365: *If I am not mistaken, these statements are inaccurate as your study did not show any significant difference in the total and direct bilirubin levels between pre-eclamptic and healthy pregnant women.*

Conclusion:

Our study revealed that serum uric acid, ALT, and AST levels were HIGHER in pre-eclamptic pregnant women compared to THOSE of healthy pregnant women, and the differences were statistically significant. As such, serum uric acid and liver function tests may be considered as early biomarkers of pre-eclampsia-related end organ damage.
