# Supplementary material for: Evaluation of serum uric acid and liver function tests among pregnant women with and without preeclampsia at the University of Gondar Comprehensive Specialized Hospital, Northwest Ethiopia
Source: PLoS One. 2022 Aug 4;17(8):e0272165. doi: 10.1371/journal.pone.0272165 (PMC9352010; doi:10.1371/journal.pone.0272165)
Supplement: Attachment — Submitted filename: Response to Reviewers.docx [file pone.0272165.s003.docx]

**June 30, 2022**

**Rebuttal letter**

Pone-d-21-30928

**Evaluation of serum uric acid and liver function tests among pregnant women with and without preeclampsia at the University of Gondar comprehensive specialized hospital, northwest Ethiopia**

**Dear Editors and Reviewers,**

We appreciate the constructive comments and suggestions for improving our manuscript. Based on the comments and suggestions, we have made corrections and modifications and provided point-by-point responses to the comments and suggestions. Please find our responses in green under the comments and suggestions presented by the academic editors in yellow. Below are our responses to each point raised by the academic editor.

Best regards!

Tadesse Asmamaw Dejenie, on behave of all authors

# Response to academic editor’s comment

**Comment 1:** Describe the definition that was used to diagnose preeclampsia in this study, were the controls having normal BP? If so please refer to controls as normotensive pregnancy.

**Author response**: We'd like to thank you for your insightful remarks and suggestions. We have selected those preeclampsia women confirmed by physicians (high blood pressure ≥ 140/90 mmHg after 20 weeks of pregnancy and having proteinuria)

**Comment 2:** Combine the information in table 1 and table 2 into one larger table with "baseline characteristics". Consider adding p value

**Author response:** Indeed, thank you very much for your suggestion to improve the clarity of our manuscript. We have combined table1 and table 2 as per your suggestion. Please check it in the revised manuscript.

**Comment 3:** Please change the column names in tables 1, 2 and 4 from "Case" and "Control" to "Preeclampsia" and "Normotensive Pregnancy"

**Author response:** Thank you so much; we've accepted your suggestions and made the necessary modifications. Please check it in the revised manuscript.

**Comment 4:** In table 2, rather than using the words: "below normal", "normal", "above normal"; provide values of BMI, SBP, and DBP. Also add proteinuria values for "cases" and "Controls", add platelet cell counts for both groups

**Author response:** Indeed, thank you very much for your suggestion to improve the clarity of our manuscript. We have modified the words: "below normal", "normal", and "above normal". We have provided values of BMI, SBP, and DBP as per your suggestion but we did not do proteinuria and platelet cell count for this study. Please find it in table 1 of the revised manuscript.

**Comment 5:** Consider deleting table 3, it does not provide extra information and becomes redundant with the information provided in table 4

**Author response:** We want to express our gratitude for your expertise. This table has been deleted. For more information, kindly check out the revised manuscript

**Comment 6:** Please review the format of the columns and the name of the columns of tables 5 and 6; it is not clear what it is being compared, and it is important for these tables to be as clear as possible, since it is the main evidence of your discussion.

**Author response:** We sincerely appreciate your insight. We removed those tables (5 and 6) since the information is already described textually. We did a post hoc (Bonferroni) test to see the SUA, ALT, and AST levels across different age and gestational age of study participants.

**Comment 7:** In table 7, consider deleting the following rows: marital status, religion, education, occupation and residence. They should not be important in order to address the difference in the lab values you are measuring.

**Author response**: Indeed, thank you very much for your suggestion to improve the clarity of our manuscript. We have accepted your suggestion and deleted the rows as per your suggestion. Please check it in the revised manuscript.

**Comment 8:** Please review your conclusion, while I agree that your "study revealed that serum uric acid, ALT, and AST levels were higher in pre-eclamptic pregnant women compared to those of healthy pregnant women, and the differences were
statistically significant."

**Author response:** Thanks, We've taken your concern into account and made the necessary modifications. Please check the conclusion section of the revised manuscript.

**Comment 9:** Please clarify if any of the patients with preeclampsia in this study were diagnosed with HELLP syndrome

**Author response:** Thank you so much for your insight. During the study period, none of the pregnant women were diagnosed with HELLP syndrome.

**Comment** 10:  Please provide information on the maternal and fetal outcomes of those with preeclampsia in this study when compared to normal pregnancy, including maternal and fetal survival, preterm delivery, birth weight, etc.

**Author response:** We really appreciate your thoughtful suggestions. A longitudinal study is required to provide information on maternal and fetal outcomes such as maternal and fetal survival, preterm delivery, and birth weight, but this is a cross-sectional study. At the time of data collection, those normotensive pregnant women came to the hospital for antenatal care follow up and those preeclampsia women were admitted to the University of Gondar hospital’s labor ward for the treatment of preeclampsia.
